# A Bacteriologic Study of the Pneumonia Occurring at Camp Pike, Ark.

**Published:** 1918-11

**Authors:** 


***A Bacteriologic Study of the Pneumonia Occurring at Camp Pike, Ark.*** By George F. Dick, M. D. *The Journal of the American Medical Association*, May 25, 1918.

Pneumonia has been one of the important diseases encountered at the Base Hospital since the opening of Camp Pike.

In 46 o/o of the cases streptococci were the predominating organism; of these, 46 o/o were non-hemolytic. These organisms formed colonies similar to the hemolytic streptococci, but had no effect on blood, neither hemolysis nor green formations being observed. The organisms were like the hemolytic streptococci as to morphology, but had more tendency to diplococcus formations, and the chains formed were short. Growth in both was flocculent and collected in the bottom of the broth. In the cases coming to necropsy, the pneumonia in which this type of organism was found resembled the ordinary pneumococcus lobar pneumonia. This type of streptococcus was found in the sputum from two cases diagnosed as influenza, and in the pus from a gangrenous appendix in greatly predominating numbers.

54 o/o of the streptococci found were hemolytic. The lesions found at necropsy in these cases were of two kinds. (1) A pneumonia similar to lobar pneumonia, due to pneumococci, but with a tendency to pus formation. In many cases this was not more marked than that sometimes observed in pneumococcus cases in others. Abscesses with areas of necrosis varied in extent, sometimes amounting to gangrene of large portions of the lung. (2) Bronchopneumonia, in which the lung swere stubbed with hard shot-like nodules which were extremely hard to the palpating fingers. Cut sections of such lungs showed areas up to 1 cm. in diameter, which were dark red and completely consolidated. This form was most commonly observed following measles. The areas in some instances coalesced to form areas amounting to a lobe in certain cases. In 25 of a series of 30 cases of mastoiditis, streptococci indistinguishable from these were isolated. The remaining five were type IV pneumococcus infections.

Both the types of streptococci were more frequently complicated by empyema than were the pneumococcus cases. In the hemolytic cases in two instances there was peritonitis.

The series is interesting on account of the high percentage of streptococcus infections, particularly those due to non-hemolytic organisms, which were also found associated with cases diagnosed as influenza, and in the pus from appendicitis.

